# Protocol for UAV fault diagnosis using signal processing and machine learning

**DOI:** 10.1016/j.xpro.2024.103351

**Published:** 2024-10-01

**Authors:** Luttfi A. Al-Haddad, Alaa Abdulhady Jaber, Nibras M. Mahdi, Sinan A. Al-Haddad, Mustafa I. Al-Karkhi, Zainab T. Al-Sharify, Ahmed Ali Farhan Ogaili

**Affiliations:** 1 Training and Workshops Center, University of Technology- Iraq, Baghdad 10066, Iraq; 2 Mechanical Engineering Department, University of Technology- Iraq, Baghdad 10066, Iraq; 3 Civil Engineering Department, University of Technology- Iraq, Baghdad 10066, Iraq; 4 Environmental Engineering Department, Al Hikma University College, Baghdad 10052, Iraq; 5 Mechanical Engineering Department, College of Engineering, Mustansiriyah University, Baghdad 10052, Iraq

**Keywords:** energy, computer sciences, environmental sciences

## Abstract

Unmanned aerial vehicles (UAVs) require fault diagnosis for safe operation. Here, we present a protocol for UAV fault diagnosis using signal processing and artificial intelligence. We describe steps for collecting vibration-based signal data, preprocessing, and feature extraction using a 3-axis accelerometer or similar sensors. We then detail the application of machine learning techniques, including deep neural networks, support vector machine, k-nearest neighbor, and other algorithms, for classifying faults. This protocol is applicable to various UAV models for accurate fault detection.

For complete details on the use and execution of this protocol, please refer to Al-Haddad et al.,^1^^,^^2^^,^^3^^,^^4^ Shandookh et al.^5^

## Highlights

• UAV fault diagnosis protocol using signal processing and AI techniques
• Vibration data collected via a 3-axis accelerometer for fault detection
• Machine learning models trained to classify UAV faults accurately
• Comparative analysis to identify the best models and features for diagnosis

## Abstract (editor highlights)

Publisher’s note: Undertaking any experimental protocol requires adherence to local institutional guidelines for laboratory safety and ethics.

## Graphical abstract

**Figure:**
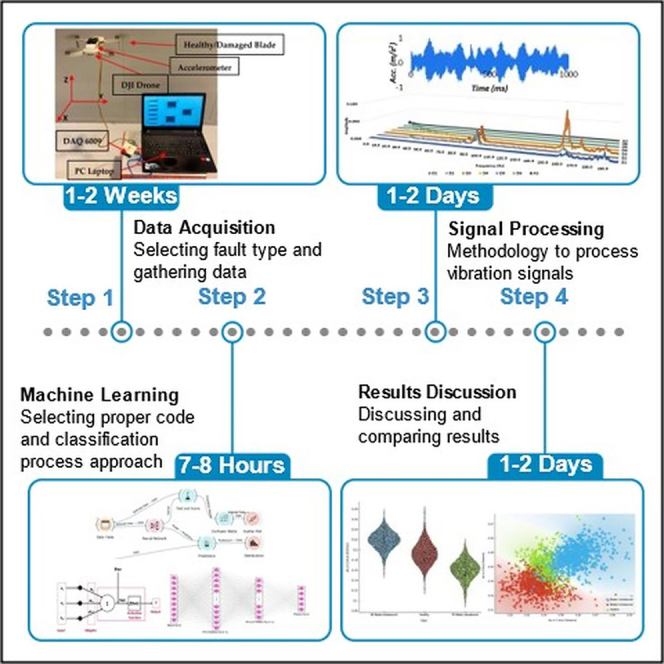

## Before you begin

### Experiment preparation

**Timing: 1–2 weeks**

1. Select Proper Drone.

Choose a suitable UAV model for the experiment. For this protocol, we select the DJI Mini Combo 2 due to its stability and ease of modification.^6^^,^^7^^,^^8^^,^^9^

2. Select Proper Data Type.
  a. Identify the type of data required for fault diagnosis. In this protocol, we focus on vibration data.
  b. Select a 3-axis accelerometer sensor capable of accurately capturing vibration data, in our works, we select the ADXL335 accelerometer.^1^^,^^2^^,^^3^^,^^4^
3. Attach Accelerometer Sensor to UAV.
  a. Secure the 3-axis accelerometer sensor to the drone frame using adhesive or mounting brackets.
  b. Align the sensor properly to capture accurate vibration data.

**CRITICAL:** Proper sensor alignment ensures the accuracy of vibration data.

4. Connect Sensor to Data Acquisition System.
  a. Connect the accelerometer sensor to the DAQ system via appropriate cables, in our setup, we use the DAQ-6009.^1^^,^^2^^,^^3^^,^^4^
  b. Ensure all connections are secure and there is no loose wiring.
5. Calibrate the Sensor.
  a. Use the DAQ software to calibrate the accelerometer sensor, in our approach, we use the LabVIEW software.^1^^,^^2^^,^^3^^,^^4^
  b. Verify calibration by conducting a test run and reviewing the data for accuracy.
6. Configure Data Acquisition System.
  a. Set the DAQ system to record vibration data in the time domain.
    i. Configure the sampling rate to capture high-resolution data.
    ii. Ensure the recording duration covers the entire hover mode operation of the UAV.
  b. Connect the DAQ system to a laptop or PC for data storage.

## Key resources table

**Table:** 

REAGENT or RESOURCE	SOURCE	IDENTIFIER
**Software and algorithms**		

LabVIEW software	National Instruments	N/A
Calibration software	National Instruments	LabVIEW

**Other**		

DJI Mini Combo 2 UAV	DJI	N/A
3-axis accelerometer sensor	Analog Devices	ADXL335
Data acquisition system (DAQ)	National Instruments	DAQ-6009
Laptop/PC with software	N/A	N/A
Adhesive/Mounting Brackets	N/A	N/A

## Step-by-step method details

### Part 1: Data acquisition

**Timing: 1–2 days**

This step involves setting up the sensor on the UAV, connecting it to the DAQ system, calibrating the sensor, configuring the DAQ system, and collecting the data.^10^^,^^11^ The description of the vibrational data acquisition is elaborated upon in Table 1.

**Figure 1:**
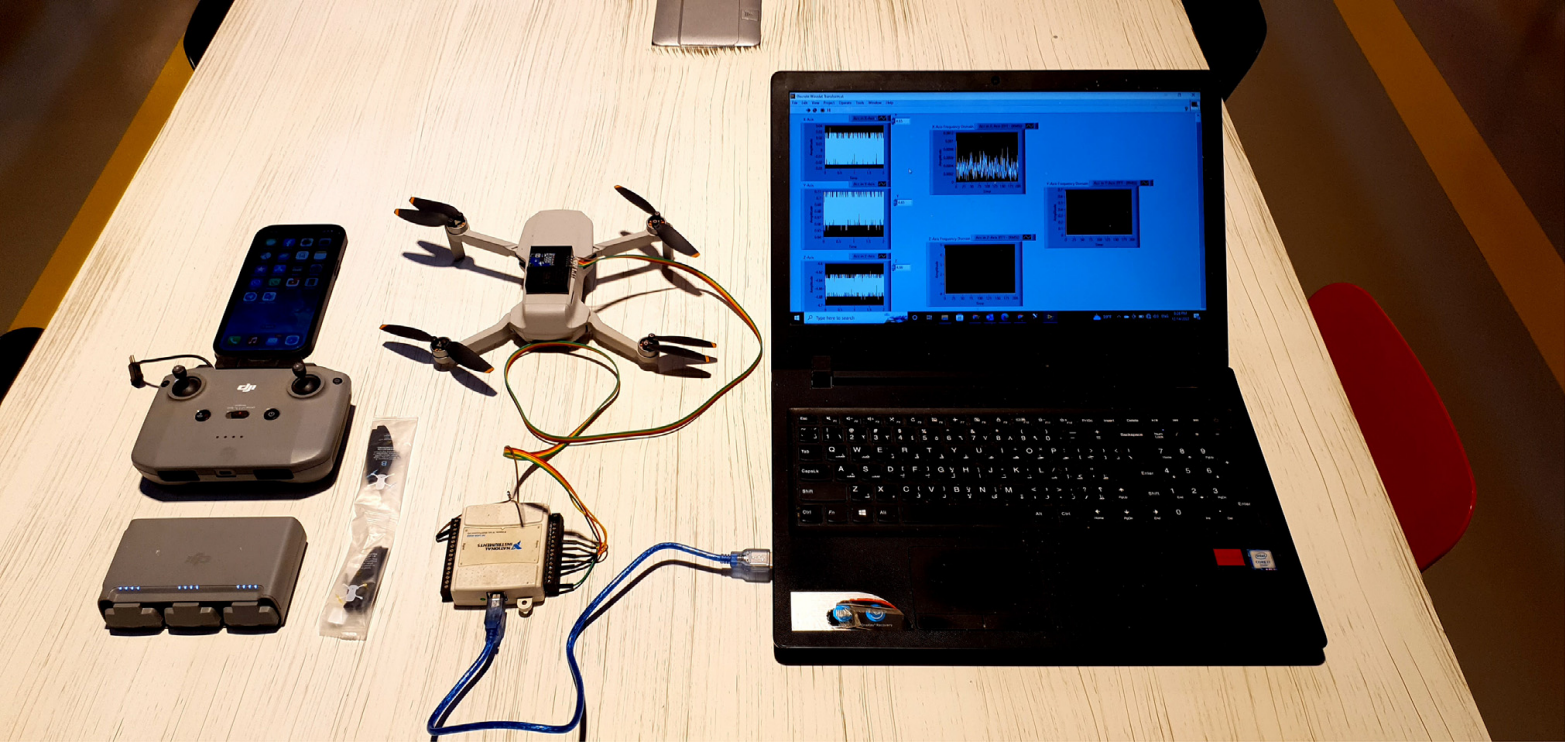
Required materials to process the current proposed protocol

1. Equipment preparation.
  a. Prepare the materials as depicted in Figure 1.
  b. Make sure that the connection works and the sensor is reading.
  c. Prepare the material to introduce the faults.
2. Identify Fault Types and Severity.
  a. Fault Types.
    i. Propeller Imbalance: Introduce a weight imbalance on one or more propellers to simulate minor and major imbalances.
    ii. Actuator Fault: Simulate actuator faults by introducing variations in the motor performance.
    iii. Blade Fault: Create blade faults by introducing cracks or other defects in the blades.
  b. Severity of Faults.
    i. Minor Fault: Introduce small imbalances or defects that cause slight deviations in vibration data.
    ii. Major Fault: Introduce significant imbalances or defects that cause noticeable deviations in vibration data.
  c. Acquire Data.
    i. Place the UAV in a controlled environment suitable for hover mode testing.
    ii. Initiate the DAQ system and start recording vibration data while the UAV is in hover mode.
    iii. Record data under different fault conditions: balanced, minor fault, and major fault.
    iv. Ensure each test is repeated multiple times to obtain reliable data, the data acquisition procedure is elaborated upon in Methods video S1.

**Table 1:** Vibration data acquisition system setup

Equipment	Specification	Amount
DJI Mini Combo 2 UAV	N/A	1 unit
3-axis Accelerometer Sensor	ADXL335	1 unit
Data Acquisition System	DAQ-6009	1 unit
LabVIEW Software	N/A	1 license
Adhesive/Mounting Brackets	N/A	Sufficient to secure sensor
Laptop/PC	N/A	1 unit
Calibration Software	Included in LabVIEW	N/A
Orange Data Mining Software	N/A	1 license

Methods video S1. Setting Up and Acquiring Vibration Data for UAV Fault Diagnosis, related to Step 2c

**Storage Conditions:** Ensure all electronic components are stored in a dry, dust-free environment at room temperature.

**CRITICAL:** Handle all electronic components with care to avoid static discharge. Ensure proper grounding when working with sensitive electronics.

***Alternatives:*** If the DJI Mini Combo 2 is unavailable, any other quadcopter can be used as an alternative UAV.^12^ If the ADXL335 accelerometer is unavailable, the MPU6050 can be used as an alternative sensor. For data acquisition, if DAQ-6009 is unavailable, a similar DAQ system that supports accelerometer inputs can be used

### Part 2: Signal processing

**Timing: 1–2 days**

This step involves preprocessing the collected vibration data to remove noise, normalizing the signal, and extracting relevant features in the time domain, frequency domain, and time-frequency domain.

3. Transfer Data to Processing System.
  a. Transfer the recorded vibration data from the DAQ system to the laptop or PC.
  b. Organize the data into appropriately labeled folders for easy access.
4. Preprocess the Data.
  a. Use signal processing software to preprocess the data, including filtering noise and normalizing the signal.
    ***Note:*** Proper filtering techniques should be applied to remove any unwanted frequencies.
  b. Visualize the preprocessed data to ensure that the noise has been effectively filtered out.
5. Extract Features in Time Domain.
  a. Calculate statistical features such as mean, variance, skewness, and kurtosis from the time-domain signal.
  b. Store the extracted features in a structured format suitable for machine learning model input.
6. Extract Features in Frequency Domain.
  a. Perform a Fast Fourier Transform (FFT) on the time-domain signal to obtain the frequency-domain representation.
  b. Calculate features such as spectral centroid, bandwidth, and power spectral density.
  c. Store the extracted features for further analysis.
7. Extract Features in Time-Frequency Domain.
  a. Apply wavelet transform or short-time Fourier transform (STFT) to the time-domain signal to obtain time-frequency representation.
  b. Calculate features such as wavelet coefficients or spectrogram statistics.
  c. Store the extracted features for further analysis.

### Part 3: Feature selection and classification

**Timing: 7–8 h**

This step involves selecting the most relevant features from the extracted feature set and classifying the data using different machine-learning models.

8. Feature Selection.
  a. Use feature selection techniques such as ReliefF or Principal Component Analysis (PCA) to identify the most relevant features.
  b. Reduce the feature set to include only the selected features for classification.
9. Classification Using Machine Learning Models.
  a. Train machine learning models such as Stochastic Gradient Descent (SGD),^1^ Deep Neural Networks (DNN),^3^ Support Vector Machine (SVM),^4^ Stacking,^2^ and k-Nearest Neighbor (kNN)^4^ using the selected features.
  b. Split the data into training and testing sets to validate the models.
  c. Evaluate the performance of each model using metrics such as accuracy, precision, recall, and F1-score.

**CRITICAL:** Proper parameter tuning during training is crucial for model accuracy

### Part 4: Comparative analysis

**Timing: 1–2 days**

This step involves comparing the performance of different machine learning models and analyzing the effectiveness of features from different domains (time, frequency, time-frequency).

10. Compare Model Performance.
  a. Analyze the performance metrics of each machine learning model.
  b. Compare the accuracy, precision, recall, and F1-score of the models to determine the best-performing model.
11. Analyze Feature Effectiveness.
  a. Compare the effectiveness of features extracted from the time domain, frequency domain, and time-frequency domain.
  b. Determine which set of features provides the highest classification accuracy and reliability.
12. Summarize Findings.
  a. Summarize the findings of the comparative analysis, highlighting the best-performing model and the most effective features.
  b. Discuss the implications of the results for UAV fault diagnosis and potential areas for further research.

## Expected outcomes

The protocol for UAV fault diagnosis using signal processing and machine learning is expected to produce comprehensive data on the vibration characteristics of the UAV under different fault conditions. It is anticipated that the protocol will yield high-quality, accurate, and reliable vibration data collected from the UAV in balanced, minor fault, and major fault conditions, which will form the foundation for subsequent analysis and model training. In addition to raw data, the preprocessing steps will result in clean, noise-free signal data ready for feature extraction. These preprocessing techniques will ensure that the vibration signals are normalized and free of unwanted noise, allowing for precise feature calculation.

From this, a comprehensive set of features will be extracted from the time domain, frequency domain, and time-frequency domain. These features will encompass various statistical measures, including mean, variance, skewness, kurtosis, spectral centroid, bandwidth, power spectral density, wavelet coefficients, and spectrogram statistics. The protocol will also result in the training of several machine learning models, such as SGD, DNN, SVM, and kNN. These models will be thoroughly evaluated based on accuracy, precision, recall, and F1 score, ensuring their effectiveness for fault diagnosis.

Furthermore, the outcomes will include a comparative analysis of the performance of these machine learning models and the features extracted from different domains. This analysis will emphasize the best-performing model and the most effective features for UAV fault diagnosis. In addition, graphical representations of the vibration data, feature extraction results, and model performance metrics will help illustrate the data and outcomes, aiding in the interpretation and validation of the protocol. Researchers utilizing this protocol can expect to produce a robust dataset of vibration signals alongside a set of reliable machine-learning models, providing valuable insights into the detection and classification of faults in UAVs and contributing to improved UAV safety and reliability.

## Quantification and statistical analysis

The analysis pipeline for this protocol involves several key steps to process, quantify, and statistically analyze the data generated. After data acquisition and preprocessing, the following methods are applied.

1. Feature Extraction: Statistical features are extracted from the time-domain signals, such as mean, variance, skewness, and kurtosis. Frequency-domain features are obtained using Fast Fourier Transform (FFT), including spectral centroid, bandwidth, and power spectral density. Additionally, time-frequency domain features are derived using wavelet transform or short-time Fourier transform (STFT), such as wavelet coefficients and spectrogram statistics.
2. Data Processing and Normalization: The preprocessed data is normalized to ensure consistency and reduce the impact of outliers, which is essential for effective machine learning model training.
3. Feature Selection: Relevant features are selected using techniques like ReliefF or Principal Component Analysis (PCA) to reduce dimensionality and improve model performance.
4. Machine Learning Model Evaluation: The dataset is split into training and testing sets for evaluating the performance of machine learning models, including SGD, DNN, SVM, and kNN. The models are trained using the training set and validated with the testing set, with performance metrics such as accuracy, precision, recall, and F1-score calculated.
5. Comparative Analysis: The performance metrics of different machine learning models are compared to determine the best-performing model. The effectiveness of features from various domains (time, frequency, and time-frequency) is also analyzed to identify the most predictive features.

## Limitations

The protocol for UAV fault diagnosis using signal processing and machine learning may have several limitations. Environmental factors such as wind, temperature variations, and humidity can affect the accuracy of vibration data collection, potentially leading to unreliable results. Mechanical limitations of the UAV, such as wear and tear of components, sensor malfunctions, or improper calibration, can also impact data quality. Additionally, the protocol’s effectiveness is dependent on the quality of the selected features and the performance of the machine learning models; suboptimal feature selection or model training may result in lower classification accuracy. Therefore, it is essential to conduct experiments under controlled conditions and regularly maintain and calibrate the equipment to ensure the validity and reliability of the results.

## Troubleshooting

### Problem 1

**Figure 2:**
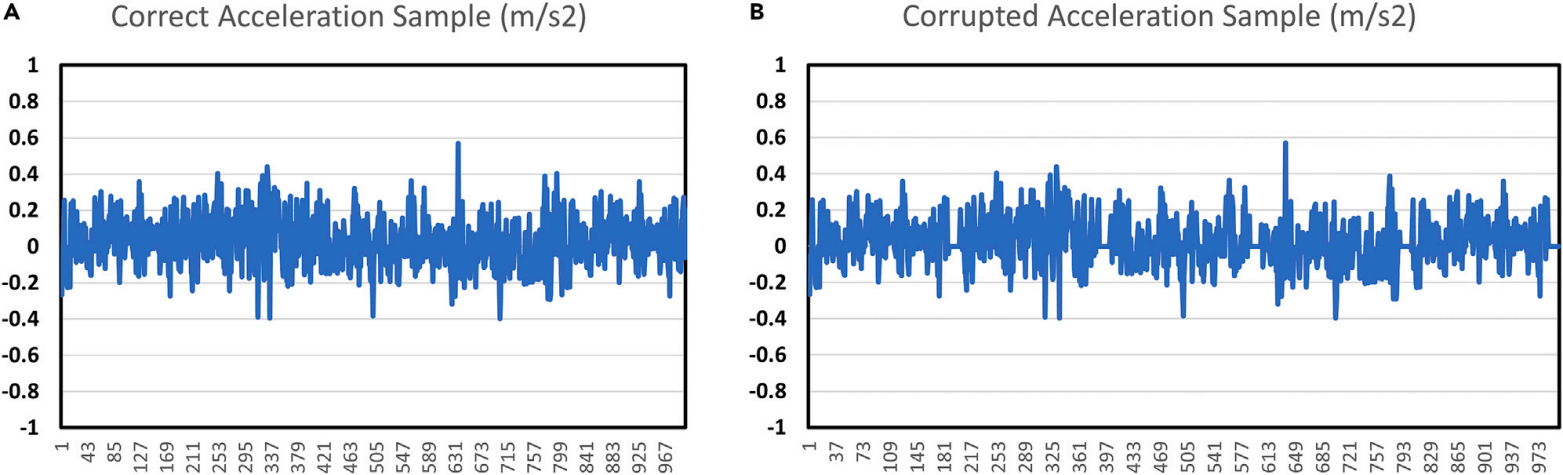
Problem 1 visualization of correct and corrupted acquired time-domain vibration signal (A) Correct signals. (B) Corrupted signals.

During the data acquisition phase, sometimes the recorded vibration signal can become corrupted due to incorrect settings in LabVIEW or other issues. This can result in unusable data, which affects the accuracy of subsequent analysis. Figure 2 illustrates an example of a corrupted signal compared to a correct signal regardless if it is under fault or no-fault conditions.

### Potential solution

To resolve this issue, ensure that all LabVIEW settings are correctly configured before starting the data acquisition. Specifically, check the sampling rate, signal filters, and synchronization settings. It is advisable to repeat the recording process twice for each test condition and verify the quality of the signal after each recording session. This approach helps to ensure that the data collected is accurate and free of corruption. If the signal appears corrupted, adjust the settings and perform the recording again until a clean signal is obtained.

## Resource availability

### Lead contact

Further information and reasonable requests for reagents may be directed to, and will be fulfilled by the lead contact, Luttfi A. Al-Haddad (Luttfi.a.alhaddad@uotechnology.edu.iq).

### Technical contact

Questions about the technical specifics of this protocol should be directed to the technical contact, Luttfi A. Al-Haddad (Luttfi.a.alhaddad@uotechnology.edu.iq).

### Materials availability

This study did not generate new unique reagents.

### Data and code availability

The datasets/code supporting the current study have not been deposited in a public repository because of privacy concerns and the proprietary nature of the UAV and data acquisition software used but are available from the corresponding author on request.

## Acknowledgments

The research received no external funding.

The authors wish to thank the University of Technology, Iraq, for providing their lab facilities. The authors would also like to thank Al-Hikma University College, Iraq, for their assistance.

## Author contributions

L.A.A.-H. was responsible for conceptualization, methodology, data collection, data analysis, writing – original draft, and supervision. A.A.J., N.M.M., S.A.A.-H., M.I.A.-K., Z.T.A.-S., and A.A.F.O. contributed to writing – review and editing.

## Declaration of interests

The authors declare no competing interests.
